# The Potential of Glucose Treatment to Reduce Reactive Oxygen Species Production and Apoptosis of Inflamed Neural Cells In Vitro

**DOI:** 10.3390/biomedicines11071837

**Published:** 2023-06-26

**Authors:** Juin-Hong Cherng, Shu-Jen Chang, Hsin-Da Tsai, Chung-Fang Chun, Gang-Yi Fan, Kenneth Dean Reeves, King Hei Stanley Lam, Yung-Tsan Wu

**Affiliations:** 1 Graduate Institute of Life Sciences, National Defense Medical Center, Taipei 11490, Taiwan; i72bbb@gmail.com (J.-H.C.); lovespring212@gmail.com (C.-F.C.); 2 Department and Graduate Institute of Biology and Anatomy, National Defense Medical Center, Taipei 11490, Taiwan; belle661011@gmail.com (S.-J.C.); u9310318@gmail.com (G.-Y.F.); 3 Department of Biomedical Engineering, Chung Yuan Christian University, Taoyuan 320314, Taiwan; 4 Laboratory of Adult Stem Cell and Tissue Regeneration, National Defense Medical Center, Taipei 11490, Taiwan; treker0315@gmail.com; 5 Private Practice PM&R and Pain Management, Roeland Park, KS 66205, USA; deanreevesmd@gmail.com; 6 The Hong Kong Institute of Musculoskeletal Medicine, Hong Kong; drlamkh@gmail.com; 7 Department of Family Medicine, The Chinese University of Hong Kong, Hong Kong; 8 Department of Family Medicine, The University of Hong Kong, Hong Kong; 9 Center for Regional Anesthesia and Pain Medicine, Chung Shan Medical University Hospital, Taichung 402, Taiwan; 10 Department of Physical Medicine and Rehabilitation, Tri-Service General Hospital, School of Medicine, National Defense Medical Center, Taipei 11490, Taiwan; 11 Integrated Pain Management Center, Tri-Service General Hospital, School of Medicine, National Defense Medical Center, Taipei 11490, Taiwan; 12 Department of Research and Development, School of Medicine, National Defense Medical Center, Taipei 11490, Taiwan

**Keywords:** glucose, treatment, neuroinflammation, reactive oxygen species, cell cycle, neural cell, in vitro

## Abstract

Neuroinflammation is a key feature in the pathogenesis of entrapment neuropathies. Clinical trial evidence suggests that perineural injection of glucose in water at entrapment sites has therapeutic benefits beyond a mere mechanical effect. We previously demonstrated that 12.5–25 mM glucose restored normal metabolism in human SH-SYFY neuronal cells rendered metabolically inactive from TNF-α exposure, a common initiator of neuroinflammation, and reduced secondary elevation of inflammatory cytokines. In the present study, we measured the effects of glucose treatment on cell survival, ROS activity, gene-related inflammation, and cell cycle regulation in the presence of neurogenic inflammation. We exposed SH-SY5Y cells to 10 ng/mL of TNF-α for 24 h to generate an inflammatory environment, followed by 24 h of exposure to 3.125, 6.25, 12.5, and 25 mM glucose. Glucose exposure, particularly at 12.5 mM, preserved apoptotic SH-SY5Y cell survival following a neuroinflammatory insult. ROS production was substantially reduced, suggesting a ROS scavenging effect. Glucose treatment significantly increased levels of CREB, JNK, and p70S6K (*p* < 0.01), pointing to antioxidative and anti-inflammatory actions through components of the MAPK family and Akt pathways but appeared underpowered (n = 6) to reach significance for NF-κB, p38, ERK1/2, Akt, and STAT5 (*p* < 0.05). Cell regulation analysis indicated that glucose treatment recovered/restored function in cells arrested in the S or G2/M-phases. In summary, glucose exposure in vitro restores function in apoptotic nerves after TNF-α exposure via several mechanisms, including ROS scavenging and enhancement of MAPK family and Akt pathways. These findings suggest that glucose injection about entrapped peripheral nerves may have several favorable biochemical actions that enhance neuronal cell function.

## 1. Introduction

Entrapment neuropathies are serious health conditions initiated by compression, stretch, or irritation of peripheral nerves that can lead to severe pain and functional disability [1,2]. Disability is often preceded or accompanied by progressive neuropathic pain [3,4]. Neuroinflammation, defined as the production of inflammatory cytokines by the affected nerves themselves, is closely associated with degenerative effects on peripheral tissue and an excessive firing rate of primary sensory neurons [5]. Evidence shows that neuroinflammation is not only a key aspect of neuropathic pain pathologies but also a feature of chronic nerve compression [3,6,7].

Treatments targeting the reduction of neuroinflammation hold promise as favorable adjunct treatments in the management of entrapment neuropathy. One emerging treatment for peripheral entrapment neuropathy is the perineural injection of glucose in water [8]. Glucose injection clinically has a prompt analgesic effect in the presence of chronic pain [9,10]. Although glucose injection induces the proliferation of chondrocytes and fibroblasts after therapeutic injection in vivo into joints and connective tissue [11,12], histologic evidence of nerve fiber infiltration/proliferation beyond natural levels has not been observed histologically due to glucose injection. Instead, an expressed goal of hypertonic dextrose injection in the treatment of tendinopathy is to reduce neovascularity and associated nerve fiber infiltration [13]. Multiple-level I–II randomized controlled trials have demonstrated consistent improvements in neural edema as measured by a reduction in the cross-sectional area of median or ulnar nerves treated with perineural hydrodissection using 5% glucose in water. This is accompanied by improved neural conduction and is proportional to improvements in pain and functional status [14,15,16,17,18]. These benefits exceed those of saline or steroid injection controls [14,15]. These observations suggest a positive effect of glucose on peripheral nerve function as a result of perineural hydrodissection, and point to a need for basic science investigation exploring the biochemical effects of glucose in the presence of the neuroinflammation that accompanies compression neuropathy.

The monosaccharide glucose (C_6_H_12_O_6_), is a primary source of energy. Its metabolism is required for adenosine triphosphate (ATP) production, which serves as the essential energy currency to support physiological brain function, the maintenance of neuronal and non-neuronal cellular function, and the generation of neurotransmitters [19]. In the inflamed nerve post-injury or during the course of repetitive injuries, inflammatory cells stimulate the excessive production of reactive oxygen species (ROS), which contribute to endothelial dysfunction and tissue damage [20]. High levels of ROS have also been associated with elevated levels of pro-inflammatory cytokines such as IL-1β, IL-6, and TNF-α, which further promote an inflammatory response autoimmunity [21]. Although dextrose injection has demonstrated clinical benefits and favorable effects on the health of connective tissue and nerves, the biochemical/genetic effects of dextrose injection remain relatively unexplored. Tumor necrosis factor-alpha (TNF-α) is widely known to contribute to the pathogenesis of neuropathic pain [22]. Our previous study demonstrated that high glucose concentrations reversed TNF-α-induced cessation of active metabolism, nuclear factor kappa beta (NF-κB) activation, and proinflammatory cytokine production [23]. It is important to further explore specifics of that anti-inflammatory effect, as that may allow for the design of studies looking at dextrose-induced changes in gene expression and in turn add to our understanding of inflammation that occurs as a result of exposure of human cells to viruses and bacteria [24,25]. In this study, our goals were to reproduce our prior finding that glucose restored normal metabolism in TNF-α-induced metabolic failure, and to extend our previous findings though in vitro analysis of subsequent glucose exposure on ROS generation, status of antioxidative and anti-inflammatory mediators acting through mitogen-activated protein kinase (MAPK) and Akt pathways, and points of impact on the cell cycle.

## 2. Materials and Methods

### 2.1. Cell Study Design

We used human SH-SY5Y neuronal cells (CRL-2266, American Type Culture Collection ATCC, Manassas, VA, USA) for this in vitro study of the effects of neuroinflammation and subsequent glucose exposure. Human SH-SY5Y neuroblastoma cells are widely used for in vitro studies of many neurological disorders, including neuroinflammatory and neurotoxicity models because SH-SY5Y cells have similar characteristics to mature nerve cells [23,26,27,28]. SH-SY5Y cells were grown in a medium containing Dulbecco’s modified Eagle medium (DMEM)/F12 Hams supplemented with 17.5 mM glucose, 100 U/mL penicillin, 100 μg/mL streptomycin (Life Technologies Inc., Carlsbad, CA, USA), 2 mM L-glutamine, and 10% (*v*/*v*) fetal bovine serum (Invitrogen, Waltham, MA, USA). Cells were incubated in a humidified atmosphere with 5% CO_2_ at 37 degrees, with the culture medium refreshed every 2–3 days. After reaching an adequate confluence, SH-SY5Y cells were seeded in 24-well plates (1 × 10^5^ cells/mL), cultured with the growth medium for 24 h, and divided into control, lesion, and treatment groups. Control group cells continued in the same growth medium for 24 h, and lesion group cells were exposed to 10 ng/mL of TNF-α for 24 h and added to the same growth medium. Treatment group SH-SY5Y cells were first cultured with 10 ng/mL of TNF-α for 24 h in baseline medium and conditions, and were then washed and immediately placed into the same medium without TNF-α and with either 3.125, 6.25, 12.5, or 25 mM of glucose.

### 2.2. Assay for Active Cell Metabolism

The presence and percentage of active cell metabolism were measured using a Cell Counting Kit 8 (CCK8) assay (A311-02, Vazyme, Nanjing, China). After 24 h of incubation, 10 µL of CCK8 solution was added to each well of each group, and incubation continued for another 45 min at 37 °C A multimode microplate reader (Thermo Fisher Scientific Inc., Waltham, MA, USA) measured absorbance at 450 nm, and was performed in triplicate. The percentage of cells with active metabolism was expressed as the number of lesion and treatment group cells with positive absorption compared with the control cell group.

### 2.3. Measurement of Reactive Oxygen Species (ROS)

Cellular ROS production was measured in triplicate with a dichlorofluorescein diacetate (DCF-DA) assay. After 24 h of incubation, 80 µM of DCF-DA was added to each well of each group and left to react for 30 min at 37 °C. Fluorescence was read at an excitation of 480 nm and emission of 535 nm using a fluorescence spectrophotometer (FLUOstar OPTIMA, BMG Labtech, Ortenberg, Germany).

### 2.4. Apoptosis Assay and Cell Cycle Analysis

To determine the points during the cell cycle where cell metabolic activity ceased, a second apoptotic assay, FITC-Annexin V/PI Apoptosis Detection Kit (BD 556405, BD Pharmingen™, San Diego, CA, USA) that preserves viability post-analysis was combined with standard cell cycle analysis by flow cytometry to investigate metabolic activity specifically during the S-phase and G2/M-phase. Following test incubation conditions, cells were collected and resuspended in a binding buffer, followed by incubation with Annexin V for 10 min at 27 °C. Next, 5 µL of propidium iodide was added prior to analysis. Cells phases were analyzed by the FAC Scan flow cytometer (Guava easyCyte 8HT Flow Cytometers, EMD Millipore, Burlington, MA, USA) and Annexin was visualized at an excitation at 488 nm. The results were analyzed by using the Guava^®^ InCyte™ Software (version 2.8; EMD Millipore). This combination test output revealed both the degree of active metabolism and the cell cycle phase during which metabolic activity ceased.

### 2.5. Luminex Assay

The cell culture supernatant was collected and each serum sample was tested in triplicate. Subsequently, a Luminex assay with MILLIPLEX^®^ Multi-Pathway 9-Plex Magnetic Bead kit was performed following the manufacturer’s instructions. The analytes available for the cell signaling multiplex assay kit were CREB (pS133), ERK (pT185/pY187), NFκB (pS536), JNK (pT183/pY185), p38 (Thr180/Tyr182), p70S6K (Thr412), STAT3 (pS727), STAT5A/B (pY694/699), and Akt (pS473). Data of fluorescence signals were analyzed using a Luminex 200 analyzer and Luminex xPONENT software (Luminex, Austin, TX, USA). 

### 2.6. Statistical Analysis

Each experiment was performed in triplicate with 6 samples/treatment (n = 6). All groups (3.125, 6.25, 12.5, and 25 mM of glucose concentrations) were compared to the lesion group. Data were obtained as mean ± standard deviation (mean ± SD) and analyzed by one-way analysis of variance (ANOVA) with a Bonferroni post hoc correction appropriate for 5 groups using IBM SPSS Statistics version 22. Statistical significance was indicated using * *p* < 0.05, ** *p* < 0.01, and *** *p* < 0.001.

## 3. Results

### 3.1. Glucose Treatment Enhances Neural Cell Survival and Reduces ROS Activity

**Figure 1:**
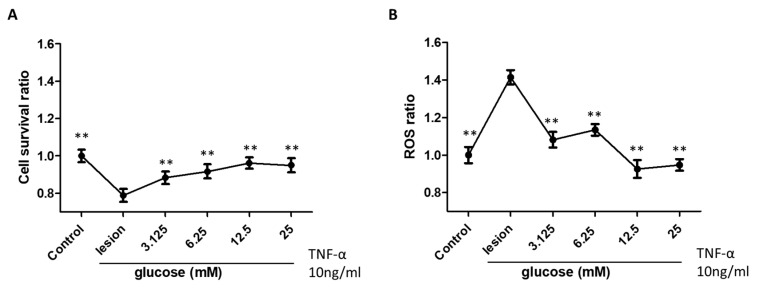
Effects of glucose treatment on cell survival (**A**) and ROS scavenging activity (**B**) of neuronal SH-SY5Y cell-induced inflammation in all groups. Each group had an n of 6, and ** represents *p* < 0.01.

The effects of glucose treatment on restoring active cellular metabolism and ROS scavenging activity of SH-SY5Y cells after induced inflammation were examined by performing CCK8 analysis and a DCF-DA assay, respectively (Figure 1). As shown in Figure 1A, compared to the control group, a significant decrease in actively metabolic cells in the lesion group was seen after exposure to TNF-α. After glucose treatment, active metabolism was significantly higher than in the lesion group not subsequently exposed to glucose. 

Exposure to TNF-α led to a 40% increase in the ROS ratio compared to the control group. The elevated ROS ratio induced by TNF-α returned to the control condition level when glucose concentration reached 12.5 to 25 mM. (Figure 1B). Although the graphs appear to favor the 12.5 mM concentrations for metabolic restoration and reduction in ROS activity, the power of this study was insufficient to confirm significant differences between various dextrose concentrations on the percentage of cells with an active metabolism.

### 3.2. Glucose Treatment Positively Regulates Cytokines with Anti-Inflammatory/Anti-Oxidative Effects

**Figure 2:**
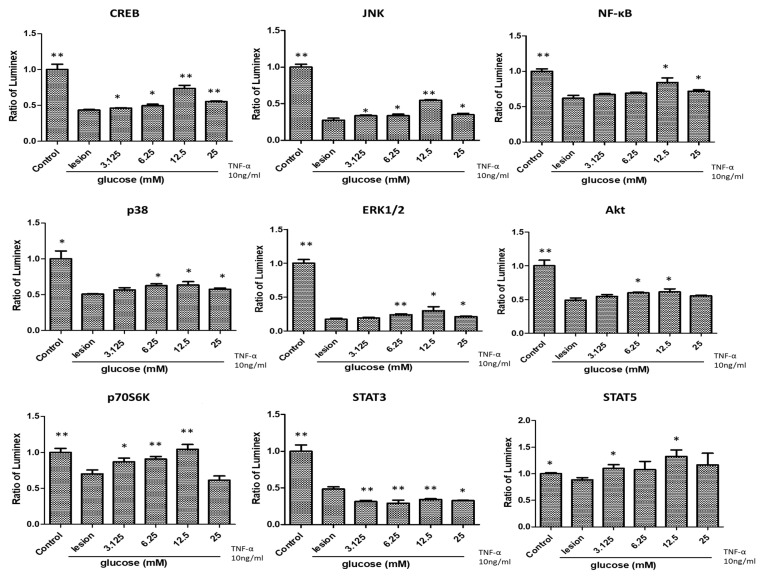
Effects of glucose treatment on the protein levels of CREB, JNK, NF-κB, p38, ERK1/2, Akt, p70S6K, STAT3, and STAT5 of neuronal SH-SY5Y cell with induced inflammation (n = 6; * *p* < 0.05; ** *p* < 0.01).

To explore the effects of glucose treatment on TNF-α- induced inflammation of SH-SY5Y cells, we performed a Luminex assay of cell-signaling anti-inflammatory/anti-oxidative biomarkers. TNF-α exposure of SH-SY5Y cells was followed by a significant reduction in all biomarkers tested except p38 and STAT5. Protein levels of CREB, JNK, and p70S6K, rose significantly after glucose treatment (*p* < 0.01), and the increase in NF-κB, p38, ERK1/2, Akt, and STAT5 approached significance after glucose treatment (*p* < 0.05), implying that glucose stimulated all these cytokines level toward a baseline (less neuroinflamed) condition (Figure 2). Dextrose 12.5 mM was associated with higher elevations than other mM levels of dextrose, but the between-group analysis was limited by size (n = 6 per group). Unlike the complete reversal of ROS upregulation seen with dextrose from 12.5–25 mM, anti-inflammatory cytokine levels were still significantly lower compared to the control group, and consistent with only a partial correction/reversal of TNF-α effects. 

### 3.3. Effects of Glucose Treatment on the Cell Cycle Regulation

**Figure 3:**
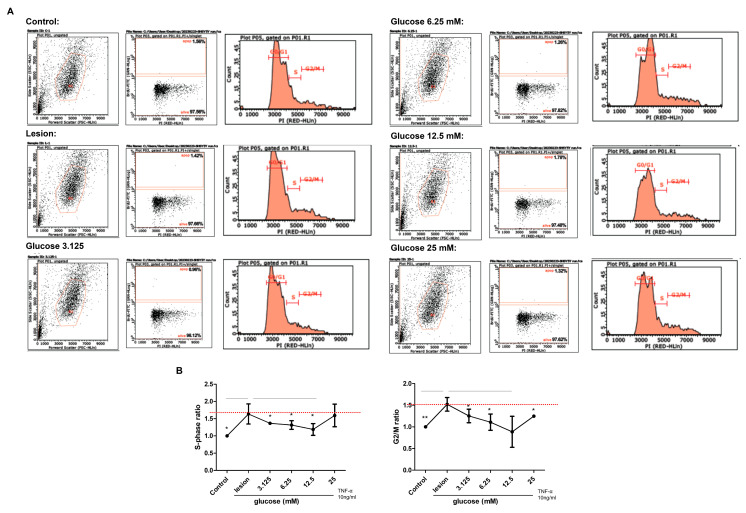
Analysis of glucose treatment effects on apoptosis and cell cycle of neuronal SH-SY5Y cell-induced inflammation in all groups. (**A**) Flow cytometry results. (**B**) Ratio of S-phase and G2/M-phase (n = 6; * *p* < 0.05; ** *p* < 0.01).

We investigated the effects of glucose treatment on the cell apoptosis and cell cycle of SH-SY5Y cells with induced inflammation using a flow cytometer (Figure 3A). The percentage of S phase in the cell cycle compared to the control group (the ratio as 1.00%) was 1.63 ± 0.29% in the lesion group, 1.36 ± 0.04% in the glucose 3.125 mM group, 1.31 ± 0.12% in the glucose 6.25 mM group, 1.18 ± 0.16% in the glucose 12.5 mM group, and 1.59 ± 0.32% in the glucose 25 mM group (Figure 3B). Overall, glucose with 12.5 mM concentration showed the smallest percentage of cell apoptosis. In the G2/M-phase, compared to the control group (the ratio as 1.00%), the percentage was 1.52 ± 0.15% in the lesion group, 1.25 ± 0.15% in the glucose 3.125 mM group, 1.10 ± 0.18% in the glucose 6.25 mM group, 0.88 ± 0.35% in the glucose 12.5 mM group, and 1.24 ± 0.02% in the glucose 25 mM group (Figure 3B). Furthermore, we found that the exposure of TNF-α to the SH-SY5Y cells notably arrested the cell cycle in both S-phase and G2/M-phase, and glucose treatment significantly lessened the proportion of cells in both phases (Figure 3B). 

## 4. Discussion

Our study shows that glucose treatment significantly enhances SH-SY5Y cell survival and reduces ROS production in vitro after induction of neuroinflammation. We found that glucose treatment can cause cells arrested in the S-phase or G2/M-phase due to an inflammatory microenvironment to exit an apoptotic state. Glucose treatment may alleviate cell apoptosis through antioxidative and anti-inflammatory actions via the MAPK family and Akt pathways. Overall, we suggest that glucose treatment potentially reduces chronic neuroinflammation and ROS activity, which is beneficial for peripheral entrapment neuropathy management.

Glucose, as an important energy substrate and an anabolic precursor, is required to sustain active cellular metabolism [29]. In glucose insufficiency conditions, some growth factors are incapable of maintaining cellular viability, resulting in cell death [29,30]. However, prolonged elevation of glucose beyond the mM levels we tested may also cause cellular dysfunction and death, depending on cell type [31]. Hence, proper glucose supplementation is necessary for maintaining nerve cell survival [32,33,34], aligning with our results. In addition, during inflammation, glucose utilization is directed more heavily toward the pentose phosphate pathway (PPP) and this action is protective against cytotoxicity caused by hydrogen peroxide, owing to a higher NADPH/NADP+ ratio and GSH level [35,36,37]. An elevation in glucose transporter 1 (GLUT1) activity also enhances ROS scavenging [38]. Accordingly, an enriched PPP glucose entry and GLUT-facilitated antioxidant are beneficial for cellular protection and ROS scavenging [39]. Perhaps useful for future study designs, subject to our power limitations, our results suggested that 12.5 mM glucose concentration may be optimal for full metabolic restoration and reduction of ROS activity in the inflammatory environment.

CREB is a stimulus-inducible transcription factor localized in the nucleus which regulates the expression of genes and intracellular processes that are important in neurons such as proliferation, differentiation, survival, neurogenesis, and neuronal plasticity [40,41,42,43]. In this study, we found that glucose treatment elevated the CREB level in SH-SY5Y cells, implying that glucose can trigger the activation of CREB, which is beneficial for nerve cell survival. The positive correlation between glucose and CREB has been observed in other studies as well [44,45]. Moreover, when CREB is activated, it regulates various adaptive changes that are substantial for neuron survival against oxidative stress or inflammation-mediated toxicity [46,47]. Our results demonstrated that glucose treatment not only enhanced CREB phosphorylation but also remarkably lessened the production of ROS.

Compared to the lesion group, we found that glucose treatment enhanced the levels of the mitogen-activated protein kinase (MAPK) subgroups including JNK (significantly), NF-κB, p38, ERK1/2, and Akt (with near significance; *p* < 0.05), which contribute to anti-apoptosis activity and cell proliferation for survival [48,49,50]. This suggests that glucose may attenuate cell apoptosis through antioxidative and anti-inflammatory actions. Interestingly, we also found that glucose treatment significantly lessened the level of STAT3 while enhancing the level of STAT5 with near significance. STATs are transcription factors activated via tyrosine phosphorylation that regulate gene transcriptions associated with various cellular processes including proliferation, differentiation, survival, and apoptosis [51,52]. Several studies have demonstrated the opposite effects of STAT3 and STAT5 activation [51,52,53]. It has been suggested that phosphorylated STAT3 is associated with the disruption of the cell cycle and cell growth [54], and in contrast, STAT5 is associated with the up-regulation of pro-survival genes [55]. Therefore, we suggest that glucose treatment affect reduces cellular apoptotic influences of STAT3, emphasizing its downstream effects through the Akt-NF-κB pathway, which helps cell survival [50]. Glucose treatment also enhances STAT5 expression, which is associated with an Akt up-regulation that encourages cell survival [56]. In addition, our study showed that the level of p70S6K was significantly increased after glucose treatment. p70S6K is known to regulate cell survival and inhibit apoptosis during increased metabolic stress as well as reduce inflammation [57,58]. Overall, our data demonstrated that glucose treatment positively stimulated anti-inflammatory, anti-apoptotic, and ROS-scavenging actions on the inflamed neural cells in vitro.

Proper cell cycle regulation is important for cellular homeostasis and cell survival. The present results reveal that exposure of TNF-α to the SH-SY5Y cells notably arrested the cell cycle in both S-phase and G2/M-phase, and glucose treatment significantly lessened the proportion of cells in both phases. These data indicated that cells with increased S-phase and G2/M-phase caused by TNF-α exposure, which is associated with damaged DNA, are prevented from undergoing mitosis by glucose treatment, offering an opportunity for repair and inhibiting the proliferation of damaged cells [59,60]. Our experimental data show that low concentrations (<12.5 mM) of glucose treatment decrease ROS production and restore cell viability, and we believe that apoptosis is not initiated due to low S- and G2/M-phase expressions. Besides inhibiting ROS production, glucose treatment causes G2/M-phase arrest; whether it will definitely lead to apoptosis is doubtful, except that we have no further evidence of apoptosis, as the main checks appear in the G1/S-phase and the G2/M-phase, in which cells are instructed to either repair DNA or undergo apoptosis when damaged. Interestingly, according to our cell molecular signaling pathways data (Figure 2), several anti-apoptotic factors such as JNK, NF-κB, p38, ERK1/2, and Akt were partially restored after glucose treatment compared to the lesion group, which means that the damaged cells are undergoing survival instead of apoptosis for cell repair, and it can considerably inhibit the inflammatory process via the MAPKs family (JNK, NF-κB, p38, and ERK1/2) and Akt pathways. This suggests that glucose treatment favors the recovery of cells arrested in the S-phase or G2/M-phase due to the influence of an inflammatory microenvironment. In addition, according to our results, glucose treatment as low as 3.125 mM promotes cell survival and reduces ROS production. As the concentration increases to 25 mM, the effect of cell survival and ROS scavenging activity will reach a limited level; and as the concentration of glucose increases, the S-phase and G2/M-phase are down-regulated, revealing a dramatically reduced level of apoptotic cells. It was also observed in the cell signaling results that glucose treatment may elevate the down-regulation of pro-inflammatory factors. Thus, we suggest that the relevant pathways of inflammatory nerve cell regeneration should be further explored. Based on the above results, we will focus on mitochondria fragmentation and ROS signaling in cell regeneration.

Basic science investigation into the effects of glucose injection around human cells in general, and nerve cells specifically, has a long history of research. The primary thrusts of that research have been in three areas: (1) the unfavorable effect of persistently elevated glucose on peripheral nerves, (2) the optimal level of glucose to use in neuronal cell culture to facilitate health and proliferation, and, more recently, (3) effects of short-term neuronal cell exposure to glucose elevation after nearby (perineural) injection of dextrose (D-glucose). In vivo animal research indicates that sustained glucose elevation for months above 19.5 Mmol (0.35%) will result in nerve fiber dropout [61]. This unfavorable influence of glucose elevation on nerves appears to be related primarily to unfavorable effects on supportive structures surrounding and within nerves such as microvasculature complications [62] or dysfunctional proliferation of soft tissue such as cell membranes [63]. Nerve cells and Schwann cells, in contrast, as high energy cells, are much more sensitive to damage from low glucose, sustaining damage rapidly with persistent mM levels below 2 mM [64], rather than high glucose levels. For example, concentrations of glucose up to 150 mM (2.7%) for 24 h did not impair the viability of Schwann cells [65] and 55 mM for 24 h did not impair ganglion nerve cell viability [66]. This differential effect of glucose on various cell types has also been recognized in the required energy component (D-glucose) in culture mediums [67]. Differences in D-glucose concentration requirements appear to differ according to the normal physiological state of the cells in question. For the maintenance and growth of endothelial cells, macrophages, and astrocytes, 5 mM d-glucose might be appropriate [68,69,70]. However, optimal survival rate and neurite growth may require much higher basal d-glucose (e.g., 25 mM; approximately 4.5 times normal blood sugar levels of 5.5 mM (0.1%)), reflecting the higher metabolic rate of neurons. The vulnerability of myelinated axons, as high energy cells, to decreases in glucose levels below 5 mM has been well described for many years and had led to a common glucose elevation level of 20 mM in culture [64]. For that reason, culture mediums of 20–25 mM D-glucose are commonly in use for the culture of neurons [71]. This is consistent with the culture medium chosen for the current study. The rapidly-growing current practice of perineural injection of dextrose to release and facilitate recovery of function in entrapment neuropathy has led to an emphasis on related basic science research. Although the rate of decrease in the concentration of dextrose after injection in soft tissue or joints may be considerable, degradation graphs are not available, and basic science research on glucose effects on nerves has relied on responses of cells in culture, typically over 24 h periods. Prolotherapy (injection to cause proliferation of connective tissue or cartilage cells) with glucose involves injection of 694–833 mM (12.5–15%) dextrose, and even perineural injection of glucose involves injection of 277 mM (5%) dextrose. Given high concentrations of glucose used in culture and the choice of cells to study that have been those less tolerant of glucose elevation, death of cells has occurred in several studies, and cell death, following by an inflammatory healing cascade, has been offered as an explanation for the therapeutic effect of glucose injection [72,73]. However, Woo et al. [74] studied dextrose concentrations as low as 55 mM (1%) and found no cell death of their studied cell (NIH-3TC; a commonly-studied fibroblast) until above 110 mM exposure for 24 h. Notably, recent studies on 10% dextrose have confirmed an absence of inflammatory cells at multiple points of biopsy after injection in rat soft tissue [75], implying a rapid degradation in glucose concentration after injection is likely in soft tissue. A rapid degradation of glucose levels after injection in vivo suggests that in vitro attempts to simulation of glucose effects on any soft tissue cell will yield inaccurate results. For that reason, beginning with our previous study [23], and continuing in the present study, we utilized much lower levels of glucose than in the previous studies [74].

This study has a few limitations. We only used SH-SY5Y cells as a cell model for neuronal inflammation. Using other neural cell types would be essential for an optimal investigation in the future. However, our findings can be valuable for providing a better understanding of the basic mechanisms of glucose treatment effects on neuroinflammation and ROS production, which are key factors in peripheral entrapment neuropathy. Another limitation is also worth mentioning. Although glucose treatment remarkably reduced the production of ROS in the current study, it is unknown how effective it would be upon comparison with an actual ROS scavenger. Given that glucose is convenient, easy to obtain, low cost, and may have considerable ROS scavenging activity, glucose may have potential as an injectate in applications related to other neuropathic or neurodegenerative disorders. Therefore, including a positive control with a real ROS scavenger in future study designs merits consideration. 

## 5. Conclusions

Glucose exposure in vitro restores function in apoptotic nerves after TNF-α exposure via several mechanisms, including ROS scavenging and enhancement of MAPK family and Akt pathways. These findings suggest that glucose injection about entrapped peripheral nerves may have several favorable biochemical actions that enhance neural cell function. 

## Data Availability

The data presented in this study are available on request from the corresponding author.
